# Role of metabolic dysfunction-associated fatty liver disease in atrial fibrillation and heart failure: molecular and clinical aspects

**DOI:** 10.3389/fcvm.2025.1573841

**Published:** 2025-04-08

**Authors:** Jamie Cheung, Bernard Man-Yung Cheung, Kai-Hang Yiu, Hung-Fat Tse, Yap-Hang Chan

**Affiliations:** ^1^ Department of Medicine, School of Clinical Medicine, The University of Hong Kong, Hong Kong SAR, China; ^2^ Department of Medicine, Shenzhen Hong Kong University Hospital, Hong Kong SAR, China; ^3^ Institute of Cardiovascular Science and Medicine, The University of Hong Kong, Hong Kong SAR, China; ^4^ Division of Experimental Medicine and Immunotherapeutics, Cambridge University Hospitals NHS Foundation Trust/University of Cambridge, Cambridge, United Kingdom; ^5^ Department of Cardiology, Royal Papworth Hospital, Cambridge Biomedical Campus, Cambridge, United Kingdom

**Keywords:** MASLD, AF, HF, cross-talk, adipokines, hepatokines, interleukins, cytokines

## Abstract

Metabolic dysfunction-associated fatty liver disease (MASLD) is a rising global health concern. In addition to direct hepatic complications, extra-hepatic complications, including cardiovascular diseases (CVD), type 2 diabetes (T2D), gastroesophageal reflux disease, chronic kidney disease and some malignancies, are increasingly recognized. CVD, including atrial fibrillation (AF) and heart failure (HF), is the leading cause of death in patients with MASLD. External factors, including excess energy intake, sedentary lifestyle and xenobiotic use, induce inflammation-related complications. MASLD, AF, and HF are associated with immune system activation, including the reprogramming of immune cells and the establishment of immune memory. Emerging evidence suggests that the heart and the liver cross-talk with each other through the diverse spectrum of autocrine, paracrine and endocrine mechanisms. Pro-inflammatory cytokines produced from the liver and the heart circulate systemically to orchestrate metabolic derangements that promote the systematic immune dysregulation in the heart-liver axis and the development of end-organ complications. Cardio-hepatic syndrome describes the clinical and biochemical evidence of hepatic dysfunction and cardiac pathology due to the interaction between the heart and the liver. Activation of inflammatory cascades, oxidative stress and immune system dysregulation underlie key mechanisms in bringing about such pathological changes. This review focuses on the current clinical and molecular evidence about the heart-liver cross-talk. It summarizes the epidemiological and pathophysiological associations of MASLD, AF and HF. In addition, we will discuss how repurposing currently available and emerging pharmacotherapies may help tackle the cardiovascular risks resulting from MASLD.

## Introduction

MASLD is a growing healthcare burden because the global prevalence of MASLD has been reported to be approximately 30% in 2019 (1). It increased by 50.4% from 25.3% in 1990–2006 to 38.0% in 2016–2019. The prevalence of MASLD in the Asian population and the Caucasian population were comparable. It was estimated in 2019 that 28.0%, 29.7% and 33.8% of the general population have MASLD in Asia Pacific, East Asia and South Asia, respectively, while the prevalence of MASLD was estimated to be 31.2% in North America and 25.1% in Western Europe (2). The global prevalence of AF was estimated to be 0.42%–0.55%. The prevalence of AF was estimated to be 0.21%–0.27% in high-income Asia-Pacific, 0.54%–0.67% in Western Europe, 0.69%–0.92% in Australasia and 0.76%–0.98% in high-income North America (3). The results of AF prevalence in Asia and the Western world were comparable. Current practice guideline suggests that modifiable risk factors for AF include obesity, sedentary lifestyle, hypertension, sleep apnea, alcoholic use, T2D and smoking (4). The prevalence of HF was estimated to be 1%–2% worldwide (5). In the United Kingdom, the age- and sex- standardized prevalence of HF was estimated to be 1.5%–1.6% (mean age at diagnosis: 76.5 ± 12.0 years) between 2002 and 2014 in a population-based study of 4 million individuals (6). The prevalence of HF was estimated to be 1.3% in the China hypertension survey study completed between 2012 and 2015, which included 22,158 study subjects ≥35 years old in the final analysis (7). Indeed, the prevalence of HF was estimated to be 1.1% in another study with 50 million individuals recruited patients from 6 provincial administrative units in 2017.

**Table 1:** Summarizes studies on the association between fatty liver and atrial fibrillation in the general population.

Authors	Country	Study designs	Year	Study subjects	Methods	Primary endpoint	Results
Kang, Min Kyu et al. (101)	Korea	Retrospective, cross-sectional, single institution study.	2010-2017	53,704 adults aged ≥ 35 years	MASLD: Ultrasonography; MFS; FIB-4 Score	Diagnosis of AF made by experienced cardiologists	Using logistic regression analysis, 59 (0.9%) out of 6,293 patients with MASLD have AF. MASLD is associated with AF (aOR: 3.84, 95% CI: 1.29–11.43, *p* = 0.016).
					AF: ECG		
Wang, Zhe et al. (102)	China	Two large-hospitals retrospective, cohort study	2019–2020	345 AF patients with NAFLD who underwent *de novo* ablation	MASLD: MFS; FIB-4 Score	Recurrence of AF, as defined by occurrence of atrial arrhythmia for more than 30s by ECG or 24 h Holter monitoring after first 3 months of ablation	134 (38.8%) out of 345 patients had recurrence of AF. Using the MFS and the FIB-4 scores, advanced liver fibrosis is independently associated with AF recurrence in multivariate cox regression model (high risk MFS = HR: 3.11, 95% CI: 1.68–5.76, *p* < 0.001; FIB-4 = HR: 3.91, 95% CI: 2.19–6.98, *p* < 0.001; intermediate risk MFS = HR: 1.85, 95% CI: 1.10–3.10, *p* = 0.020; FIB-4 = HR: 2.08, 95% CI: 1.27–3.41, *p* = 0.003).
					AF: ECG or 24 h Holter monitoring		
Käräjämäki, Aki J et al. (103)	Finland	Prospective cohort study using national health registry	1990–1993	958 patients middle-aged hypertensive subjects and age- and sex- matched control subjects	MASLD: ultrasonography	Incident AF, as data retrieved from national health registry	Using adjusted cox regression analysis, MASLD is associated with risk of AF (HR: 1.88, 95% CI: 1.03–3.45), after adjusting for age, sex, study group, diabetes status, BMI, waist, drinking alcohol, smoking, serum ALT, SBP and other cardiac factors.
					AF: ECG; based on ICD-10 code from hospital discharge registry		
Van Kleef, Laurens A et al. (104)	Netherland	Prospective cohort study	2009–2014	5,825 study subjects who joined the abdominal ultrasound program	MASLD: ultrasonography	Incident AF, as measured by ECG	MASLD was not associated with prevalent or incident AF, while liver stiffness was associated with prevalent AF (OR 1.09 per kPa, 95% CI 1.03–1.16).
					AF: ECG		
Boeckmans, Joost et al. (105)	Germany	5-year prospective follow-up population-based proteomic study	2012–2017	11,509 adults between 35- and 74 years old	MASLD: FIB-4 score; FIB- = 4 score < 1.3 = low risk and FIB-4 ≥ 1.3 intermediate and high risk	Changes in the level of CXCL10 that is associated with prevalent AF	Using multivariate linear regression model, after adjusting for age, sex, smoking, arterial hypertension, diabetes mellitus, obesity, dyslipidemia, coronary artery disease and congestive HF, the standardized log (FIB-4 index) was significantly related with CXCL10 (β-estimate 0.160 with 95% CI: 0.127–0.194, *p* < 0.0001), suggesting an association between MASLD and AF.
					AF, as determined by, CXCL10, biomarker level		
Long, Michelle T et al. (106)	United States	Community-based, longitudinal cohort study	2002–2005	2,122 study subjects from the Framingham Heart Study Offspring and Third Generation cohorts	Liver fat: CT	Incident AF, as measured using ECG at each doctor's visit	No significant association was found between MASLD and prevalent AF and no association was found between MASLD and incident AF after 12 year of follow-up
					AF: ECG at each study visit, as well as ECGs and Holter monitor reports from physician and hospital records		

AF, atrial fibrillation; CT, computed tomography; chemokine (C-X-C motif) ligand 1; ECG, electrocardiogram; FIB-4 score, fibrosis-4 score; HF, heart failure; MASLD, metabolic-associated dysfunction fatty liver disease; NAFLD, non-alcoholic fatty liver disease fibrosis score; MFS, fibrosis score.

**Table 2:** Summarizes studies on the association between fatty liver and heart failure in the general population.

Authors	Country	Study designs	Year	Study subjects	Methods	Primary endpoint	Reported correlation
Liu, Xiao et al. (108)	United States	Cross-sectional Study	2011–2018	19,695 study subjects ≥20 years old	Non-invasive fibrosis markers: MFS, FIB-4 and APRI score	Self-reported diagnosis of HF based on the “Monetary Choice Questionnaire”	Using the logistic regression analysis, a significant association is found between advanced liver fibrosis and HF prevalence (FIB-4 OR: 1.15, 95% CI: 1.07–1.23; MFS OR: 1.42, 95% CI: 1.23–1.64; APRI OR 1.44, 95% CI: 1.15–1.81), after adjusted for cardiometabolic confounders.
Hydes TJ, Kennedy OJ et al. (109)	United Kingdom	Longitudinal, prospective cohort study	2006–2010	413,860 participants from the UK biobank	Non-invasive fibrosis markers: MFS, FIB-4 and APRI score	Incident HF, defined as an ICD-9 (428.0–428.9) or ICD-10 (I50.0–I50.9)	Liver fibrosis is associated with an increased risk of hospitalization or death from HF (multivariable adjusted high-risk MFS score HR, 1.59; 95% CI 1.47–1.76; *p* < .0001; FIB-4 HR, 1.69; 95% CI, 1.55–1.84; *p* < .0001; APRI HR, 1.85; 95% CI, 1.56–2.19; *p* < .0001; combined fibrosis scores HR, 1.90; 95% CI, 1.44–2.49; *p* < .0001) using cox regression analysis.
Roderburg, Christoph et al. (110)	Germany	Retrospective cohort study	2005–2020	173,966 patients ≥ 18 years	Patient records with MASLD and HF were retrieved from Disease Analyzer database	Cumulative incidence of HF (ICD 10: I50)	MASLD is associated with a higher risk of developing HF (HR: 1.34, 95% CI: 1.28–1.39, *p* < 0.001) using cox regression model.
Wu, Shouling et al. (111)	China	Prospective cohort study	2006–2020	96,576 study subjects aged ≥18 years old	Hepatic steatosis was diagnosed by abdominal ultrasonography	HF, as defined using ICD 10 code I50.x	Using cox regression model, mild-MAFLD (HR: 1.27, 95% CI: 1.16–1.39) and significant-MAFLD (HR: 1.45, 95% CI: 1.31–1.63) are associated with a higher risk of HF in all participants, after adjusting for confounders.
Ohno R, Kaneko H, Suzuki Y, et al. (113)	Japan	Retrospective observational study	2005–2021	aged >20 years, 3,279,918 individuals	Fatty liver disease was defined as FLI of >30	HF as defined using ICD codes (ICD-10: I500, I501, I509, and I110) and AF (ICD-10: I480, I481, I482, I483, I484, and I489).	After multivariate adjustment, the HRs for HF are 1.20 (95% CI: 1.18–1.23) for the metabolic dysfunction group, 1.24 (95% CI: 1.19–1.30) for the FLD group, and 1.73 (95% CI: 1.69–1.76) for the MAFLD group compared with the non-FLD/non–metabolic dysfunction group. After multivariate adjustment, the HRs for AF are 1.13 (95% CI: 1.08–1.19) for the metabolic dysfunction group, 1.13 (95% CI: 1.04–1.23) for the FLD group, and 1.51 (95% CI: 1.46–1.57) for the MAFLD group compared with the non-FLD/nonmetabolic dysfunction group.

APRI, aminotransferase to platelet ratio index; CI, confidence interval; FIB-4 score, Fibrosis-4 score; FLI, fatty liver index, as defined by the Japan research group; HF, heart failure; HR, hazard ratio; MFS, non-alcoholic fibrosis score; OR, odds ratio.

**Table 3:** Summarizes evidence on repurposing existing cardio-protective therapeutic agents to treat MASLD to prevent AF and HF.

Authors	Study drug	Country	Study designs	Year	Study subjects	Methods	Primary endpoint	Results
Harrison, Stephen A et al. (128)	Resmetirom	245 sites, 15 countries	Phrase 3, Randomized, Controlled Trial	2019–2021	966 patients	MASH status is measured by liver biopsy	MASH resolution as assessed by liver biopsy at week 52 (reduction in MASLD activity score ≥ 2 points; or at least one stage improvement with no worsening of the MAFLD activity score).	25.9% of the patients in the 80 mg resmetirom group and 29.9% of those in the 100 mg resmetirom group, as compared with 9.7% of those in the placebo group (*P* < 0.001 for both comparisons with placebo) achieved the goal of MASH resolution with no worsening of fibrosis. 24.2% of the patients in the 80 mg resmetirom group and 25.9% of those in the 100 mg resmetirom group, as compared with 14.2% of those in the placebo group (*p* < 0.001 for both comparisons with placebo) achieved the goal of at least one stage with no worsening of the MASLD activity score.
Schreiner, Andrew D et al. (119)	Statins	United States	Retrospective Cohort Study	2012–2021	1,238 patients with MASLD	Statin use and MASLD diagnosis were retrieved from EHR data and fibrosis score was calculated based on lab results.	Time to a high-risk advanced fibrosis risk. (High risk for advanced fibrosis was defined as a FIB-4 ≥ 2.67 during follow-up)	In the cox regression analysis, statin intensity is associated with lower risk to progress to high-risk FIB-4 category (moderate statin intensity HR: 0.60, 95% CI: 0.42–0.84, and high statin intensity HR: 0.61, 95% CI: 0.42–0.88), after adjustment for sex, race, marital status, smoking status, body mass index, hypertension, diabetes, cardiovascular disease, hypothyroidism and chronic kidney disease.
Haukeland, John Willy et al. (121)	500 mg metformin daily	Norway	4 university hospital, double-blind, placebo-controlled RCT	2004–2007	24 study subjects (without T2D) in each arm	Assessment: liver biopsy	Changes in liver steatosis between the index biopsy and the second biopsy.	No significant differences between treatment with metformin or placebo were observed for changes in liver steatosis, assessed either histologically or by CT, NAS-score, liver transaminases or on markers of insulin resistance or inflammation.
						Treatment: 6-month 500 mg metformin daily		
Armstrong, Matthew James et al. (123)	1.8 mg Liraglutide daily	United Kingdom	Multicentre, double-blinded, randomised, placebo-controlled phase 2 trial	2010–2013	23 study subjects with biopsy-confirmed steatohepatitis (stratified by T2D status) in each arm	Assessment: improvement in liver histology from baseline to end of treatment.	Resolution of definite non-alcoholic steatohepatitis with no worsening in fibrosis	Patients with diabetes and treated with liraglutide had better improvement (RR: 4.3, 95% CI: 1.0–17.7, *p* = 0.019), when compared to placebo.
Cheung, Ka Shing et al. (124)	10 mg empagliflozin daily	China	Investigator-initiated double-blinded, placebo controlled RCT	2021–2022	49 study subjects with hepatic steatosis and without T2D in each arm	Assessment: MRI-PDFF for hepatic steatosis Treatment: 52-week 10 mg empagliflozin	Changes in MRI-PDFF	The empagliflozin group had a greater reduction in median MRI-PDFF compared to the placebo group (−2.49% vs. −1.43%; *p* = 0.025), with a nonsignificant trend of resolution of hepatic steatosis (44.9% vs. 28.6%; *p* = 0.094), using the intention-to-treat analysis.
Belfort, Renata et al. (117)	45 mg pioglitazone daily	United States	Double-blinded, placebo-controlled RCT	2002–2004	55 patients with impaired glucose tolerance or T2D and liver biopsy-confirmed steatohepatitis	Assessment: MRI-PDFF and liver biopsy	Improvement in histological presentation for steatohepatitis	The pioglitazone group had improvement in steatosis (*p* = 0.003), ballooning necrosis (*p* = 0.02), and inflammation (*p* = 0.008), when compared to the placebo group, when compared to placebo group.
						Treatment: 6-month 45 mg pioglitazone		

CT, computed tomography; EHR, electronic health record; MASLD, metabolic-associated fatty liver disease; MRI-PDFF, magnetic resonance imaging-derived proton density fat fraction; RCT, randomized controlled trial; T2D, type 2 diabetes.

A two-way relationship exists between the heart and the liver; many patients with MASLD have inflammation-related atrial and ventricular myopathy. Unhealthy lifestyles, such as excessive eating behaviour, sedentary lifestyle and inhalation and ingestion of xenobiotics, alter the normal physiological pathways. Obesity commonly results from an energy imbalance between energy intake (excessive nutrition) and energy expenditure (physical inactivity) (8). Our team previously reported that liver stiffness is a prognostic value and an integrative correlate of right heart function (9), and liver stiffness is associated with different stages of HF (10, 11). Our team also reported that liver stiffness is associated with tricuspid regurgitation (TR) (12), of which, TR is associated with AF (13). In addition, in a comprehensive bioinformatics and Mendelian randomization (MR) analysis, a positive causal relationship between MASLD and HF has been demonstrated (14). However, no significant causal association has been found between MASLD and AF in MR analysis (15). The exact underlying mechanisms explaining these observations have not been reported yet. Therefore, we will discuss the potential two-way relationship of MASLD, HF and AF in this review.

Mild metabolic inflammation is a hallmark feature of obesity-related complications, including MASLD, AF and HF (16). It has been hypothesized that ectopic fat formation is accompanied by the release of adipokines, which can activate the immune system and increase pro-inflammatory mediator production (17, 18). AF and HF can be caused directly by the excessive increase of epicardial adipose tissue or by myocardial inflammation and oxidative stress paracrine modulators indirectly (19). Apart from adipokines, hepatokines from the liver, cardiokines from the heart, extracellular vesicles and immune cells are produced to cross-talk among multi-organs (20–22). Cardio-hepatic syndrome is the terminology used to describe the systematic illness caused by the interaction between the heart and the liver (23). Liver injuries are commonly observed in patients with decompensated heart failure. Cardiac cirrhosis is the consequence of the hemodynamic imbalance in the circulatory system. Right heart failure, valvular disease, severe pulmonary hypertension, cor pulmonale, biventricular heart failure, pericardial diseases, cardiac tamponade and constrictive pericarditis cause, increased cardiac filling pressures and preload, low cardiac output and venous congestion; thus therefore, increase pressures in the hepatic sinusoids (24). Such elevation of retrograde pressure causes an increase in liver enzymes, which may lead to acute hepatocellular necrosis.

Approximately 25% of the cardiac output flows through the liver from the portal vein and hepatic artery mix, then through sinusoids to interact with hepatocytes, and then leaves the liver through the hepatic veins to the right side of the heart through the inferior vena cava (25). MASLD is characterized by an elevating influx of free fatty acids (FFAs) to the liver (26). FFAs are either covalently bound in the triacylglycerol core of circulating lipoproteins or attached to the plasma albumin to be transferred from the capillary lumen to the cardiac muscle cell (27). Fatty acid oxidation is an essential source of adenosine triphosphate (ATP) (28). However, an imbalance between the lipid uptake and lipid utilization in the heart causes increased intracellular lipid accumulation, including triglycerides (TGs), diacylglycerols (DAGs), ceramides and cholesterols. DAGs and ceramides are cardiotoxic mediators. In addition, excess FFAs lead to increased transcription of systemic inflammatory mediators that travel to the heart through circulation. Systematic inflammation causes a dramatic influence on the epicardium, leading to lipolysis and further release of fatty acids, resulting in further reactive inflammation (29).

The inflammatory process reduces the production of adiponectin (30). It increases the expansion of epicardial adipocyte mass to further elevate the synthesis of pro-inflammatory adipokines [e.g., leptin, tumour necrosis factor (TNF)-alpha (α), interleukin (IL)-1beta (β), IL-6]. Inflammatory changes in multiple locations within the heart lead to electro-anatomical remodelling. In addition, the persistence of chronic inflammation leads to enhanced oxidative stress, gradual mitochondrial damage and pro-inflammatory factor and pro-thrombogenic molecule production, which in turn is associated with histologic progression, myocardial stress and structural derangements. Consequently, fibrosis of the adjoining myocardium occurs as a result.

Hepatic injury is a common observation in patients with HF. The arterial buffer response is the ability of the hepatic artery to compensate for the portal blood changes. Portal hypertension (31), due to increased portal inflow, is the classical feature of liver fibrosis (32). The fibrotic nodule formation of the liver leads to splanchnic arteriolar vasodilatation, which is the consequence of vasodilator spill-over and vasoconstrictor over-sensitivity, resulting in a dysregulated bile acid physiological pathway (33). Bile acids are known to impact the heart because G protein-coupled bile acid receptor-1, nuclear bile acid receptor, and the Farnesoid X-activated receptor are found to be expressed in the cardiomyocytes and vascular endothelial cells (34). Bile acids alter the functional, structural, and electrophysiological changes in cirrhotic myopathy by changing myocardial cells' β-adrenoceptor density and membrane fluidity. In addition, portal hypertension leads to leakage in the gut barrier, allowing bacterial translocation and gut microbiota dysbiosis. The bacterial translocation leads to the release of nitric oxide (NO) and pathogen-associated molecular patterns (PAMPs) to vasodilate arteries, causing hyperdynamic circulation. Along with insulin resistance, hepatic inflammation, pro-inflammatory mediation and cirrhosis-associated immune dysfunction, the damaged liver stimulates the release of TNF-α and IL-1β, leading to circulatory and cardiac malfunction.

Elevation of adipokines enhances the infiltration of macrophages, injury of microvascular structures and stimulation of pro-fibrosis in the underlying tissues (35). The maladaptive malformation of the epicardium forms fibroblasts that thicken the epicardial atrial and ventricular muscles. Ischemia-reperfusion injury is characterized by hypoperfusion-induced hypoxia. The low-flow states exacerbate liver congestion through increasing systemic venous pressures. The hepatic ischemia-reperfusion injury in HF is presented with early activation of Kupffer cells, late activation of polymorphonuclear cells, intracellular calcium overload, oxidative stress, mitochondrial damage and disruption of liver microcirculation. Liver cirrhosis leads to expanded plasma volume, lowered systemic vascular resistance, and increased cardiac chamber dimensions and pressures, resulting in high output HF. The microvascular rarefaction, cardiac fibrosis and decreased ventricular distensibility of ventricular myocardium are the hallmark features of HF (36).

Furthermore, the left ventricular pressure of HF increases pulmonary pressure, which leads to pulmonary congestion, dyspnea and tachypnea, fluid transudation and pulmonary crackles (37). As the peripheral circulation is obstructed, nutrient malabsorption and renal and hepatic dysfunctions result. In the long term, compensatory mechanisms in neurohormonal systems are activated to cause liver congestion, ascites and edema, further deteriorating and enlarging the heart (38).

## The role of pro-inflammatory cytokines in liver lesion and cardiac injury

Adipocyte hyperplasia and hypertrophy are associated with increased production of inflammatory cytokines, for example, TNF-α, IL-1α, IL-1β, IL-6, IL-17, IL-18 and IL-37 (39). TNF-α is the first adipocytokine, and it is a soluble mediator. It has been observed that the lack of TNF-α is associated with improved insulin sensitivity (40, 41). The elevation of TNF-α leads to systemic low-grade inflammation and macrophage recruitment that contribute to MASLD. TNF-α induces apoptosis, angiogenesis and thrombogenesis in both myocytes and endothelial cells, implicating the development of heart disease (42, 43). IL-1α is normally expressed in the epithelial and mesenchymal cells of the myocardium, and its level is elevated during cardiac injury (44). IL-1β is the major circulating subtype of IL-1 and is upregulated in the disease state. Both IL-1β and IL-18 are produced primarily from immune cells (45). 1l-1β mainly induces inflammation through IL-6 signaling, while IL-18 stimulates the release of IL-17. IL-17 is used as a marker to identify clusters of differentiation (CD)4+ T helper cells (46), and IL-17 works through nuclear factor-kappa beta (κβ) and mitogen-activated protein kinase (MAPK) pathways to regulate pro-inflammatory gene transcription. As fibroblasts are the primary target of IL-17, fibrosis is induced (47). Although most cytokines induce harmful effects, several cytokines have protective effects. For example, IL-37 and IL-38 reduce the levels of pro-inflammatory cytokines (48–50). Among all the interleukins mentioned above, IL-37 is particularly interesting because IL-37 is a newly identified cytokine discovered in 2000. It belongs to the IL-1 family cytokine. At the tissue level, IL-37 is mainly expressed in low amounts in various locations, including tonsils, esophagus, placenta, melanoma, breast, brain, colon, prostate, lung, liver, heart and bone marrow. At the cellular level, IL-37 is expressed in epithelial cells, keratinocytes, renal tubular epithelial cells, monocytes, activated B cells, plasma cells, dendritic cells (DCs), macrophages and CD4+ Tregs (51).

IL-37 helps to shift cytokine expression from pro- to anti-inflammation. IL-37 inhibits both innate and adaptive immunological responses. Therefore, it has been used as a prognostic marker in various autoimmune diseases. IL-37 exerts an immunosuppressive effect to promote macrophage polarization intracellularly and extracellularly. IL-37 is produced as a precursor in the cytoplasm of the cell. Inflammation stimulates the production of the IL-37 precursor and activation of the caspase-1, leading to maturation of the IL-37. IL-37 is then translocated into the nucleus to suppress the transcription of proinflammatory genes through binding to mothers against decapentaplegic homolog 3 (SMAD-3). Alternatively, IL-37 is delivered outside of the cell and binds to the IL-18Ra chain to recruit Toll/IL-1R (TIR)-8 for anti-inflammatory signalling transduction. The expression of IL-37 is inducible, and its upregulation helps to suppress toll-like receptor (TLR) agonists and pro-inflammatory cytokines.

Indeed, the systemic level of IL-37 differs before and after liver injury, suggesting IL-37 plays a significant role in restoring cell metabolism and repairing liver lesions. IL-37 inhibits hepatitis, liver injury, liver fibrosis and hepatocellular carcinoma. The exact mechanism of action of IL-37 is still unknown. Interestingly, macrophages with IL-37 inhibit the proinflammatory cytokines induced by TLRs because IL-37 is identified as a natural inhibitor of congenital inflammation and the innate immune response. Besides liver injury, IL-37 is also involved in cardiovascular diseases, including atherosclerosis, myocardial infarction (MI) and ischemia/reperfusion injury, because IL-37 reduces inflammatory responses in negative feedback mechanisms. In addition, IL-37 inhibits transcription of genes for several pro-inflammatory cytokines and chemokines, such as TNF-α and IL-6. However, the role of IL-37 in AF and HF has not been investigated yet.

## Pathophysiological paradigm

**Figure 1:**
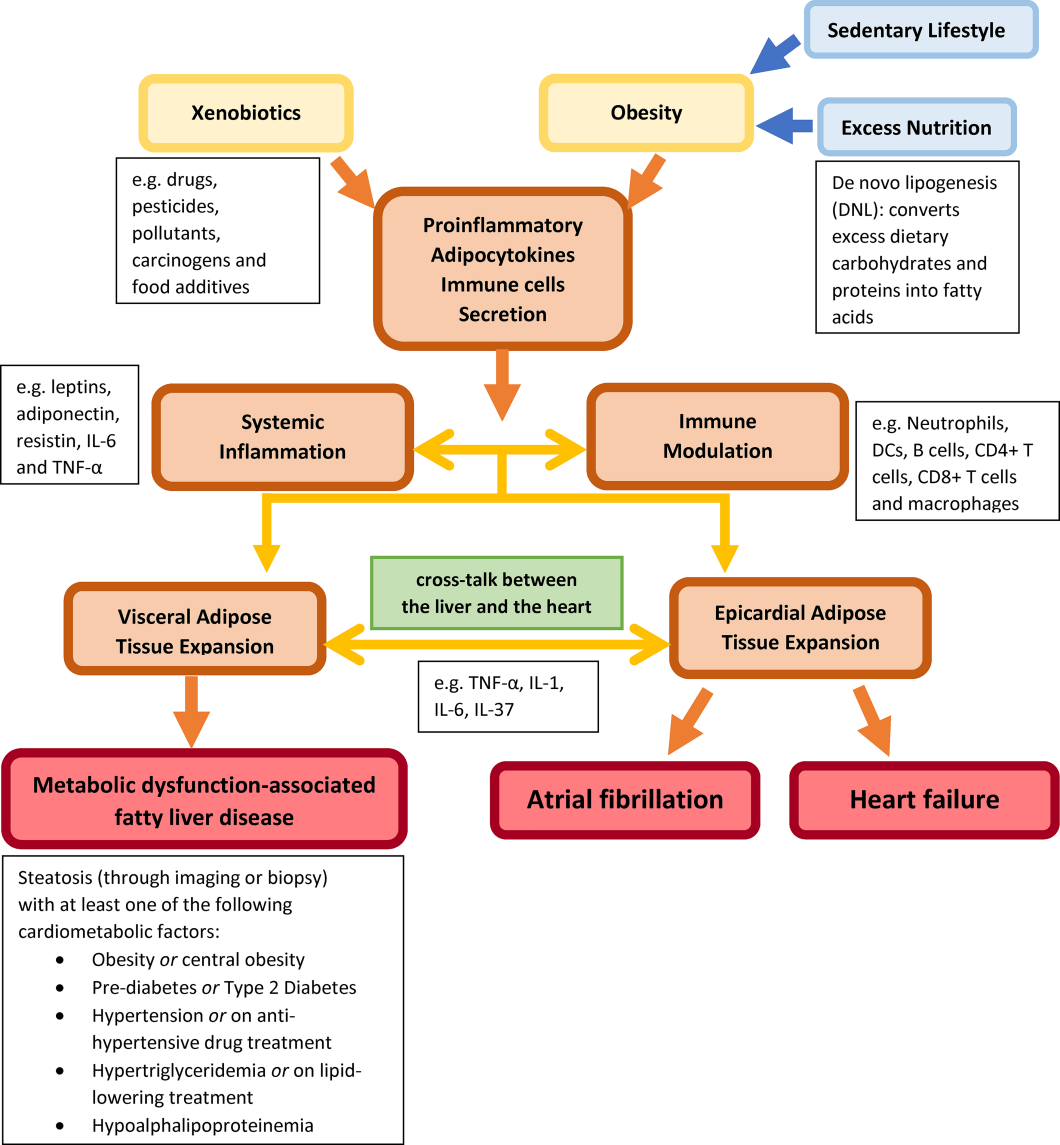
Describing external factors (xenobiotic use and obesity) inducing systemic inflammation and immune modulation through circulating pro-inflammatory adipokines and immune cells between the heart and the liver to induce MASLD, AF and HF.

The consumption of excess macronutrients and the use of xenobiotics, including drugs, pesticides, pollutants, carcinogens, and food additives, induce obesity and obesity-related inflammation and cardiometabolic diseases due to circulatory pro-inflammatory cytokines, adipokines and hepatokines over-secretion (Figure 1). Signalling molecules serve as transmitters for cell-to-cell communications, which allow cross-talk among cardiometabolic organs. The systemic inflammation further induces visceral and epicardial adipose tissue expansion, respectively. Correspondingly, the localized inflammatory effects of excess adipose tissues in the liver and the heart lead to MASLD, AF and HF.

## Hepatokines, adipokines, and cardiokines communicate with each other in autocrine, endocrine and paracrine manners

In recent years, interest has been growing in organokines, including hepatokines, adipokines and cardiokines, which act on the secreting tissue (autocrine) or the adjacent tissue (paracrine) to produce localized effects. These organokines are direct communicators between white adipose tissue (WAT), liver and heart to cause MASLD, AF and HF. Hepatokines are hormones the liver produces that interfere with the endocrine-dependent relationship; each hepatokine has different functions. Fetuin A increases visceral adipose tissue (VAT) and causes pancreatic-β cellular damage and insulin resistance. Fetuin B promotes insulin resistance and T2D development and is associated with the severity of steatosis. Activin E significantly reduces adipocyte lipolysis and fat accumulation to mitigate obesity and MASLD (52). In contrast, another hepatokine, tsukushi, reduces cholesterol efflux capacity and cholesterol-to-bile acid conversion in the liver in response to MASLD (53).

It has been well-recognized that the heart is a secretory and an endocrine organ (54). Cardiokines are hormones produced from different parts of the heart, including myocytes, fibroblasts, endothelial and vascular cells that help to maintain metabolic homeostasis under physiological conditions, including regulating lipolysis, adipogenesis, energy expenditure and thermogenesis against cold exposure and adipokine biosynthesis. Brain natriuretic peptide (BNP), TNF- *α* and angiotensin II (Ang II) are classic examples of cardiokines. Recently, more and more cardiokines have been discovered using the gene expression array screening big data analytical technique. It is important to note that paracrine and autocrine signalling within the heart is the most common form of physiological communication that directs the normal functioning of the heart. However, under cardiac stress, e.g., MI and HF, cardiokines are secreted to compensate for the cardiac disturbance. Using the genomic approach, it is estimated that 1,000–2,000 kinds of cardiokines exist (55), and about 30–60 types are validated in experimental settings. Cardiokines are either secreted in the classical secretory pathway in the endoplasmic reticulum (ER)-associated ribosomes or the nonclassical secretory pathway in the cytosolic ribosomes that must be transported across the plasma membrane independently of the ER-Golgi pathway. Mediator complex subunit 13 (MED13) is a cardiokine that helps gain fat mass and body weight, improves systemic insulin sensitivity and glucose tolerance, increases systemic energy expenditure, regulates WAT gene expression, and promotes fatty acid oxidation (56).

Interestingly, fibroblast growth factor (FGF)-21 could be classified as hepatokine, adipokine, or cardiokine. Under normal physiological conditions, the liver secretes most of the FGF-21 to regulate metabolism, including fatty acid β-oxidation, ketogenesis, and gluconeogenesis (57). In patients with HF, the cardiomyocytes secrete FGF-21 (58–60). FGF-21 controls glucose homeostasis, insulin sensitivity and ketogenesis, reduces body weight gain and influences adipokines synthesis and secretion. FGF-21 prevents the induction of proinflammatory pathways in the heart and reverses cardiac hypertrophy development. It inhibits cardiomyocyte apoptosis after MI incidence, attenuates pathological myocardial remodelling and reduces infarct size. It stimulates lipid oxidation and thermogenesis to cause browning of WAT and modulate the pathogenesis of heart failure (61). In addition, FGF-21 also exacerbates the development of hepatic steatosis (62). As mentioned earlier, the heart is an endocrine organ in the atrial natriuretic peptide (ANP), and brain natriuretic peptide (BNP) is transmitted via endocrine mechanisms to act as “antagonists” to regulate renal electrolyte and water excretion in the renin-angiotensin-aldosterone system and to promote vascular cell growth and vasodilatory tone.

Interestingly, ANP and BNP are recently found to act on the natriuretic receptors on adipose tissues and have a strong lipolytic effect. Apart from cardiokines, adipokines are hormones produced from the adipose tissues. They regulate lipid metabolism, insulin sensitivity, appetite-rewarding response, fibrogenesis and liver fat accumulation. To date, many adipokines have been discovered, and each has different roles. Resistin leads to an increase in fat mass. Adiponectin (63) and leptin (64) increase fatty acid oxidation, which helps to enhance insulin sensitivity and reduce lipid storage, respectively. Lipocalin 2 regulates inflammation and transports fatty acids, leading to low-grade systemic inflammation, vascular remodelling, and atherosclerotic plaque instability (65). Vaspin protects the vascular tissues from fatty acid-induced apoptosis because it reduces pro-inflammatory cytokines and improves glucose intolerance and insulin sensitivity (66). At the same time, visfatin produces adipocyte inflammation, insulin resistance and pancreatic β cell dysfunction (67).

## Recent breakthrough in molecular mechanisms of heart-liver cross-talk

Multiple studies reported the potential underlying molecular mechanisms involved in the liver-heart cross-talk. In an animal study that analyzed the transcriptomic and functional genetic data, coagulation factor (FXI) protects against diastolic dysfunction, fibrosis and inflammation in mice with heart failure (68). FXI activates the bone morphogenetic protein (BMP)—suppressor of mothers against decapentaplegic (SMAD)1/5 pathway in cardiomyocytes. In the mice that overexpressed FXI, 124 genes related to circadian rhythm, cardiac muscle contraction, inflammation, focal adhesion, the phosphatidylinositol 3-kinase (PI3K)-Akt pathway and insulin signalling pathway are altered. FXI protease activates the resulting growth factor fragment by cleaving the BMP7 proprotein. The plasma of 21 patients with heart failure was examined to translate the results from bench to bedside. It was found that FXI is inversely correlated with E/e' ratio in all participants, suggesting that FXI protects against diastolic dysfunction in patients with heart failure. Another study investigated the role of Ly6C^hi^ monocytes in mice with MI to promote hepatic fibrosis (69). MI alters the immune regulation in mice with metabolic-associated steatohepatitis (MASH). Increased intrahepatic recruitment of CD45+ immune cells and increased proportion of CD11b^+^F4/80^+^Tim4^−^ macrophages, but a decreased amount of CD11b^+^F4/80^+^Tim4^−^ macrophages is observed. Moreover, the MASH-associated macrophages (NAM) and hepatic F4/80^+^Trem2^+^ are further increased in mice after MI. In addition, CD11b^+^F4/80^int^Ly6C^hi^ immune cells are increased in mice with MASH after MI, and these immune cells can be merged into CCR2^+^ monocytes in the liver of mice with MASH. These CCR2^+^Ly6C^hi^ immune cells are further elevated in mice with MASH after MI. Subsequently, circulating CD45^+^CD115^+^ cells are higher than the mice without MASH. Periostin (POSTN), a glycoprotein predominantly secreted by osteoblasts with multifunction in modulating the cell fate determination, proliferation, inflammatory responses, fibrosis and tumorigenesis, is found to be overexpressed in mice with accelerated hepatic pathological progression of MASH. POSTN activates c-Jun NH2-Terminal Kinase (JNK) 1/2 and inhibits peroxisome proliferator-activated receptors (PPAR)α to accumulate lipids in hepatocytes; it also promotes hepatic fibrosis by activating integrin β3 signalling. Lastly, the mediating effect of serum follistatin-like protein 3 (FSTL3) is found to be associated with increased liver fibrosis risk in patients with MI and T2D (*n* = 1,424), using the Gene Expression Omnibus (GEO) analysis.

## Complex immune interactions between the heart and the liver

Activation of innate and adaptive immune response pathways is one of the essential key mechanisms to provoke cardiac adverse remodelling because immune cells modulate the inflammatory response to cause ischemic cardiomyopathy (70). During the innate response phase, the inflammatory immune cells, including neutrophils and monocytes/macrophages, infiltrate the damaged heart area to initiate an early healing response (71). These neutrophils polarize into phenotypes, including N1 and N2, to infiltrate the damaged heart region. Apart from secreting inflammatory cytokines and chemokines, cardiomyocytes respond to stress stimuli by producing damage-associated molecular patterns (DAMPs) and PAMPs to expand resident immune cells and recruit bone marrow-derived immune cells from the circulation. The polarized macrophages are activated to cross-talk with surrounding cells, such as platelets (72). Through toll-like receptors (TLRs) signalling pathways, DAMPs and PAMPs activate nucleotide-binding oligomerization (NOD)-like receptor family pyrin domain-containing protein 3 (NLRP3) inflammasome to activate immune cells and fibroblasts to promote pro-hypertrophic and pro-fibrotic signalling. As a result, the infiltration of leucocytes and necrotic cardiomyocytes to repair the infarcted area through scar formation to maintain cardiac integrity. In addition, CD4+ T cells are activated to facilitate the cardiac remodelling further. In the adaptive immunity activation phase, antigen-presenting cells—DCs and distinct antigen-specific receptors on T cells are involved. DCs loaded with a heart-specific self-peptide induce T-cell mediated myocarditis through myeloid differentiation primary response 88 (MyD88)/IL-1 signalling in the bone marrow compartment (73).

Besides the heart, the liver is also a highly vascularized organ in which capillary-like vessels known as sinusoids provide space to allow immune cells to detect blood-borne and gut-derived pathogens (74, 75). The liver contains a lot of immune cells, which circulate or reside in sinusoids or parenchyma. Immune cells that temporarily regulate the hepatic system include natural killer (NK) cells, γβ T cells, CD4+ and CD8+ αβ T cells, monocytes, B cells, invariant NKT (iNKT) cells, mucosal-associated invariant T (MAIT) cells and DCs. In contrast, immune cells that are long-lived in the liver include Kupffer cells, CD8+ tissue-resident memory T (T_RM_) and type 1 innate lymphoid cells (ILC1s). Different immune cells have different functions, and these immune cells modulate the pathogenesis of MASH. For example, neutrophils are responsible for the early infiltration during MASH, which causes inflammation, liver injury, and fibrosis. The number of DCs is increased during MASH to activate inflammation and liver injury. B cells are heterogenous and pro-inflammatory and can subdivided into two major lineages: B1 and B2. B1 cells secrete “natural” antibodies as a response to innate immunity.

In contrast, B2 cells are activated in secondary lymphoid organs and receive help from CD4^+^ T helper (T_H_) cells to generate antigen-specific, high-affinity antibodies. The decreased number of B2 cells is associated with reduced MASH-associated liver fibrosis. In addition, the liver contains a large amount of immunoglobin (Ig)A-producing plasma cells, and IgA is associated with advanced hepatic fibrosis in patients with MASH. Apart from contributing to the MASH, CD4^+^ T cells cause fatty acid-induced death during MASH, the conventional CD8^+^ T cells, including the CXCR6^+^ subset, and T_H_17 cells, including the CXCR3^+^ subset, contribute to fibrosis, steatosis and liver injury that eventually lead to hepatocellular carcinoma (HCC) because CD8^+^ T cells are involved in direct hepatocyte killing.

Bioinformatics analyses and experimental validation have been performed to identify and examine common signature genes of immune cells underlying the pathogenesis association between MASLD and HF (76). Immune markers, CD164 and CCR1, are found to have diagnostic values for MASLD and HF. However, bioinformatics analyses must be validated against experimental lab data or real-world data because studies are published to indicate that genomic-based data may not be accurate and translational (77–79). Nevertheless, casual relationships and shared genetic architecture are found between heart and liver diseases (80). However, real-world data to trace the potential molecular mechanisms involved have been lacking to support the findings from genomic studies.

## Metabolic dysfunction-associated fatty liver disease

MASLD is a histological spectrum of disease ranging from steatosis, which is a relatively benign, lack of inflammation and reversible stage of MASLD, to steatohepatitis, which is an active and inflammatory stage of MASLD as fat has been infiltrated into the hepatocytes (81). Hepatic steatosis is defined by the presence of 5% or more triglyceride content in the liver. Steatohepatitis is characterized by macro-vesicular steatosis, hepatocellular ballooning, lobular inflammation and pericellular fibrosis, and it may result in irreversible and fatal conditions, such as cirrhosis and hepatocellular carcinoma. The golden standard to identify MASLD is liver biopsy; however, it induces pain; therefore, it is not widely accepted by most patients. Apart from liver biopsy, magnetic resonance imaging-proton density fat fraction (MRI-PDFF) and transient elastography (TE) are ultrasonography commonly used to identify patients with hepatic steatosis and steatohepatitis non-invasively. TE is increasingly used to detect liver steatosis and steatohepatitis due to its high sensitivities and specificities to detect MASLD and its high patient acceptance rate due to its non-invasive nature (82). Apart from having hepatic steatosis, MASLD is diagnosed by meeting one of the five following cardiometabolic risk factor criteria, including obesity, hyperglycemia, hypertension, hypertriglyceridemia, or hypoalphalipoproteinemia. These cardiometabolic risk factors are known to intensify the severity of MASLD, leading to liver-related complications.

In addition to MRI-PDFF and TE used to identify patients with hepatic steatosis, metabolic-dysfunction associated fibrosis score (MFS), fibrosis-4 (FIB-4) and aspartate aminotransferase (AST) to platelet ratio index (APRI) are non-invasive scores that utilized routine laboratory blood test results to calculate the liver fibrosis risks. MFS is the MASLD fibrosis score that predicts liver fibrosis based on age, body mass index (BMI), platelets, albumin, AST/alanine aminotransferase (ALT) and impaired fasting glucose (IFG) diabetes status (83). The FIB-4 index is commonly used as a surrogate marker to predict liver stiffness, and it is calculated using age, AST, platelet count and ALT levels in the blood (84). However, the threshold to cut off the FIB-4 index to define advanced liver fibrosis has not yet been well established. Researchers usually divide the population into tertiles to describe patients with low-, intermediate- and high-risk liver fibrosis based on the FIB-4 index. APRI has a simple formula that only utilizes AST and platelet count to calculate the fibrosis risk.

Besides these conventional fibrosis scores, two surrogate markers were validated in the human clinical trials in 2022–2023 to identify patients with steatohepatitis for risk stratification. In a study, liver histological assessments were performed on 252 patients [median age: 30.0, interquartile range (IQR): 25.0–37.0, 42.1% men, median BMI: 38.2, IQR: 33.4–44.5 kg/m^2^] with obesity, the result of the liver histological assessments was compared with the liver and blood levels of thrombospondin-2 (TSP2), and it was observed that TSP2 hepatic and blood serum expressions were elevated in patients with metabolic syndrome and steatohepatitis (85). In another cohort study that examined the feasibility of using soluble CUB domain-containing protein 1 (sCDCP1) as a non-invasive steatohepatitis biomarker in obese individuals, 191 out of 489 study subjects (median age: 31, IQR: 25–36, 43.1% men) were found to have steatohepatitis and the use of sCDCP1 was found to be highly correlated with the use of liver biopsy to detect steatohepatitis, as the area under the receiver operating characteristics (AUROC) achieved 0.838 (95% CI: 0.789–0.887) (86).

In the 1970s, the “two-hit hypothesis” was proposed to describe the formation of MASLD. The first hit refers to the inability of the liver to metabolize the excess energy substrates, causing accumulation of adipose tissue and insulin resistance within the liver. In contrast, the second hit refers to activating the inflammatory cascades and fibrogenesis from the adipose tissue to cause liver fibrosis (87). However, it has been becoming more evident recently that the “two-hit hypothesis” is too simplistic to describe the etiology of MASLD, as MASLD is indeed a more complex disease, as multiple hits are involved. These multiple hits include metabolic disturbances, lipotoxicity, insulin resistance, chronic inflammation, fibrosis and gut microbiota imbalance. These factors, all together, act synergistically to implicate MASLD (88).

Obesity is the most common cause of insulin resistance. Insulin is a pancreatic hormone produced to regulate glucose metabolism in response to the post-prandial glucose excursion, and it plays a significant role in limiting hepatic gluconeogenesis and promoting glucose absorption into the peripheral tissues (89). However, when an individual fails to utilize insulin to uptake glucose into muscle and adipose tissues, the individual is said to have insulin resistance. It is because adipokines, cytokines, chemokines, excess lipids and toxic lipid metabolites from adipose tissues alter the regular insulin physiological pathway. Insulin resistance leads to activated lipolysis in the adipose tissue and the release of FFAs into the bloodstream. The circulating FFAs stimulate hepatic *de novo* lipogenesis (DNL) and TGs accumulation in hepatocytes and impair inhibition of adipose tissue lipolysis (90). DAGs are the precursor for the synthesis of TGs (91). In case excessive DAGs are sensed, DAGs activate protein kinase C epsilon, decreasing the proximal insulin signalling. Excess glucose enters hepatocytes via insulin-independent pathways that further stimulate DNL. DNL triggers excess carbohydrates into fatty acids, which are then esterified to form TGs stored in the liver and VAT.

Compared to WAT, VAT is more metabolically active. It contains more preadipocytes with less differentiating capacity accompanied by inflammatory and immune cells. It is more insulin-resistant, resulting in increased lipolytic activity and FFAs production. The visceral fat depot drains directly into the portal circulation. The portal drainage of VAT delivers FFAs to the liver with DNL in both hepatocytes and adipocytes in the liver, resulting in a vicious cycle. As a result, with insulin resistance, the FFAs alter the formation and secretion of adipokines and inflammatory cytokines, including IL-37, IL-1β, IL-6 and IL-10, resulting in systemic inflammation. Lipotoxicity is another factor for MASLD, where excessive amounts of FFAs, free cholesterol and other lipid metabolites cause oxidative stress-associated mitochondrial dysfunction, reactive oxygen species (ROS) production and endoplasmic reticulum stress-related mechanism activation. The co-existence of MASLD and CVD comorbidities in many patients indicates a shared underlying pathophysiology between them (92). Arrthymia, atherosclerotic heart disease and HF are all commonly observed in patients with MASLD.

## Atrial fibrillation and heart failure

Altered ion channels, autonomic nervous system abnormalities, inflammation, calcium handling abnormalities, dysregulated renin-angiotensin-aldosterone system (RAAS), transforming growth factor- β (TGF-β) over-secretion and oxidative stress are believed to contribute to the development of MASLD (93). Similar cytokines may exert pathophysiologically relevant effects on the heart. Firstly, disturbed movements of ions on the transmembrane of the cardiomyocytes and atrial fibroblast of the heart cause AF by affecting the cardiac systole and diastole. Multiple reasons could cause abnormal movement of cardiac ions, such as genetic mutations. Some individuals with mutated genes affecting the sodium ion channels have reduced sodium current density, shortened refractory period, increased re-entry of cardiomyocytes and slowed atrial conduction velocity. In contrast, some individuals with mutated calcium channel genes have dysregulated flow of calcium ions in the L-type calcium channel, leading to a longer effective refractory period in cardiomyocytes. Indeed, some individuals with a reduced L-type calcium channel have a shortened refractory period that may trigger AF. Secondly, sympathetic and parasympathetic nerves innervating in different heart locations, especially those in the posterior wall of the left atrium, are known to cause AF. Parasympathetic nerves are predominant in the heart and cause heterogeneous electrophysiological changes in the atria. Thirdly, the cytoplasmic pattern-recognition receptor that recognizes pathogens and cellular damages, called inflammasome, is a key sensor to induce apoptosis in the innate immune system. In the state of obesity, inflammasome causes obesity-induced atrial arrhythmogenesis. Moreover, inflammatory cytokines, including IL-6, are associated with incident AF and TNF-α, which are associated with calcium disturbance. TGF-β promotes pro-fibrotic effects of Ang II-induced fibrosis under the RAAS pathway. It has been noted that Ang II is chemotactic for inflammatory cells that activate nicotinamide adenine dinucleotide phosphate (NADPH) oxidase to induce oxidative stress. Such activation results in a positive feedback loop between the RAAS pathway component to cause chronic inflammation. Aldosterone is the end-product of RAAS, which can activate the mineralocorticoid receptors on the cardiomyocytes and cardiac fibroblasts to decrease the nitric oxide availability to cause cardiac inflammation, hypertrophy and fibrosis to impair vasculature. HF, stroke, chronic kidney disease, cognitive dysfunction and dementia are all possible consequences of AF.

Cardiotoxic xenobiotics, such as cocaine and anthracycline, can induce cardiotoxicity and HF acutely or chronically (94, 95). According to the American Heart Association, the structural pathological changes of the heart in patients with HF include reduced left or right ventricular systolic function, reduced ejection fraction and reduced strain, ventricular hypertrophy, chamber enlargement, wall motion abnormalities and valvular heart disease (96). Cardiomyocyte loss, for example, myocyte loss due to MI and valvular disease with apoptosis due to overload, hypertrophy, and necrosis (97), is the hallmark feature of HF. The primary underlying pathophysiological mechanisms of HF are volume overload and pressure overload. The permanent neurohumoral activation of the RAAS leads to volume overload (98) and pressure overload, impairing ventricular relaxation and increasing ventricular stiffness to elevate the ventricular filling pressure further and dilate the ventricular chamber, resulting in eccentric cardiac structural remodelling. In addition, the release of vasoactive peptides stimulated through activation of RAAS increases the preload and circulatory fluid. It enhances afterload via the sympathetic α1-receptor vasoconstriction mechanism in the kidney, vasculature and skeletal muscle. The neurohumoral activation, vasoconstriction, increased oxidative stress and imbalance of nitric oxide and energy bioavailability result in endothelial dysfunction. Also, RAAS initiates the β1-mediated intracellular cyclic adenosine monophosphate (cAMP), which increases calcium to cause α1-receptor mediated peripheral vasoconstriction and chronotropic effect. Stimulation of the carotid sinus and aortic arch baroreceptors leads to enhanced release of vasopressin and antidiuretic hormone (ADH) to cause fluid retention that worsens HF (99).

## MASLD is associated with AF and HF in the general population

### Atrial fibrillation

MASLD is a multisystem disease that increases the risks of not only liver-related outcomes and extra-hepatic cancers, but patients with MASLD are also associated with a moderately elevated risk of incident AF independently (Table 1) (100). In a retrospective, cross-sectional cohort study consisting of 53,704 study subjects completed by a Korean hepatology group from January 2010–December 2017 (101), 6,293 study subjects were identified to have MASLD. Among these 6,293 study subjects, 59 (0.9%) were diagnosed with AF by experienced cardiologists. The mean age of those who have AF were older (mean age: 64.6 ± 8.7 vs. 52.0 ± 9.3 years), more likely to be men (79.7% vs. 58.0%), more obese (BMI: 26.6 ± 2.8 vs. 25.2 ± 2.6 kg/m^2^) and more likely to have T2D (22.0% vs. 9.4%). Analyzing the results using the high cut-off values of FIB-4 score by logistic regression analysis, AF is independently associated with advanced liver fibrosis (OR: 3.84, 95% CI: 1.29–11.43, *p* = 0.016) after sequential adjustment for gender, presence of diabetes, hypertension, obesity, total cholesterol, triglyceride, high-density lipoprotein, low-density lipoprotein, albumin, gamma-glutamyl transferase and high-sensitivity C-reactive protein.

In another retrospective study conducted from January 2019–December 2020 in two large hospitals in China, a total of 345 AF patients (mean age: 62.1 ± 9.4 years, 66.4% men) who had *de novo* ablation were recruited and followed up for a year to study the recurrence rate of AF (102). Among all patients recruited, 38.8% had recurrence. The results were then further analyzed using the multivariate Cox regression model. Patients with advanced liver are independently associated with AF recurrence in multivariate Cox regression model (high risk: MFS, HR: 3.11, 95% CI: 1.68–5.76, *p* < 0.001, FIB-4, HR: 3.91, 95% CI: 2.19–6.98, *p* < 0.001, intermediate risk: MFS, HR: 1.85, 95% CI: 1.10–3.10, *p* = 0.020, FIB-4, HR: 2.08, 95% CI: 1.27–3.41, *p* = 0.003).

In the prospective age- and sex-matched control follow-up study with 958 study subjects (MASLD mean age: 52 ± 6 years, 58% men and non-MASLD mean age: 51 ± 6 years, 43% men) recruited in Finland from 1990–1993 (103), patients were tracked for AF diagnosis with a median follow up time of 16.3 years (range 0–19 years), using the National Death Registry and/or hospital discharge registry. It was found that 14.9% (*n* = 37) of subjects with MASLD at baseline had been diagnosed with AF. In the adjusted multivariate Cox regression model, MASLD is associated with the risk of AF development (OR: 1.88, 95% CI: 1.03–3.45, *p* = 0.001).

In a large-scale prospective cohort study that took place in the Netherlands between 2009 and 2014 (104), with 5,825 participants included (mean age 69.5 ± 9.1 years, 49.2% men), liver stiffness is significantly associated with prevalent AF (OR 1.09 per kPa, 95% CI: 1.03–1.16, *p* = −0.002) using logistic regression adjusting for age, sex, alcohol consumption, smoking, education level, prevalent HF, prevalent cardiac heart disease and the individual component of metabolic syndrome, spleen size and inferior vena cava diameter and alanine aminotransferase (ALT).

In a prospective follow-up general population cohort study (105) including 11,509 participants (mean age of 54.0 ± 11.1 years, 48.7% men), the C-X-C motif chemokine ligand 10 (CXCL10), which is known to be the interface linking between liver fibrosis and AF, was studied in a 5-year prospective follow-up study. A total of 15,010 study subjects were enrolled, and 11,509 study subjects (mean age 54.0 ± 11.1 years, 48.7% men) were included; CXCL10 was found to be the inflammatory nexus between liver fibrosis and prevalent AF using a multivariate linear regression model. After adjusting for age, sex, smoking, arterial hypertension, diabetes mellitus, obesity, dyslipidemia, coronary artery disease and congestive HF, the standardized log (FIB-4 index) is significant relativity CXCL10 (β-estimate 0.160 with 95% CI:0.127–0.194, *p* < 0.0001).

Overall, the results of these studies show positive significant associations between MASLD and AF, suggesting treating MASLD, which is a relatively benign disease, may prevent or treat AF. However, no statistically significant associations are found between MASLD, incident AF and prevalent AF in a longitudinal cohort study conducted in the United States, which a total of 2,122 participants (mean age: 59.0 ± 9.6 years) with baseline data obtained in 2002–2005 were included in the final analysis (106). The insignificant results may be because of the relatively smaller sample size. As well, computed tomography is not the golden standard to examine MASLD, the sensitivity and specificity of CT to detect mild steatosis are not high, 57% and 88%, respectively (107).

### Heart failure

MASLD is associated with HF (Table 2). In a cross-sectional study conducted using the National Health and Nutrition and Examination Survey from 2011–2018 (108) with 19,695 study subjects enrolled (median age: 47.0, IQR: 33.0–61.0, 48.6% men), advanced liver fibrosis was determined by three non-invasive liver fibrosis scores, namely FIB-4, MFS and aspartate aminotransferase to platelet ratio index (APRI). FIB-4 and MFS are commonly used to assess liver fibrosis, and APRI utilized AST and platelet count only, which is less accurate when compared to FIB-4 and MFS. Using the logistic regression analysis, significant associations between advanced liver fibrosis and HF prevalence (FIB-4 OR: 1.15, 95% CI: 1.07–1.23; MFS OR: 1.42, 95% CI: 1.23–1.64, APRI OR 1.44, 95% CI: 1.15–1.81) are found, after adjusting for cardiometabolic confounders.

In a longitudinal prospective UK biobank cohort study conducted during 2010–2017 (109), 413,860 study subjects aged 40–69 (median age: 58 years, IQR: 13 and 45.5% men) were identified during the health screening to have liver fibrosis. These patients were included in the final multivariate Cox regression analysis that looked at the association between liver fibrosis and incident HF after a median of 10.7 years. A total of 12,527 cases of hospitalization or death from HF were recorded. Using multivariate Cox regression analysis, MFS (HR: 1.59, 95% CI: 1.47–1.78, *p* < 0.0001), FIB-4 (HR: 1.69, 95% CI: 1.55–1.84, *P* < 0.0001), and APRI scores (HR: 1.85, 95% CI: 1.56–2.19, *p* < 0.0001) are significantly associated with the HF incidence.

In a retrospective study conducted from 2005–2020 (110), 86,983 adult study subjects were matched with a cohort of 86,983 patients without NAFLD using propensity risk score. The mean age of these 173,966 patients was 57.2 ± 14.3 years, and 52.6% were men. MASLD is associated with a higher risk of developing HF (HR: 1.34, 95% CI: 1.28–1.39, *p* < 0.001) using a Cox regression model.

In a prospective cohort study (111) conducted in China, with the baseline data obtained between 2006 and 2007 and followed up for 14.0 years until 2020, 96,576 study subjects were recruited in the final analysis. Using Cox regression analysis, mild-MAFLD (HR: 1.27, 95% CI: 1.16–1.39) and significant-MAFLD (HR: 1.45, 95% CI: 1.31–1.63) are associated with a higher risk of HF in all participants, after adjusting for confounders. Interestingly, interactions by sex and age were observed for the associations mentioned above. It was found that MAFLD-associated HF decreased with age in men and women, but the sex difference this week was only present in study subjects younger than 45 years old. The result suggests that women of reproductive age may have different female sex hormones produced that alter their HF risk. This study's results aligned with the previous findings (112) that women are more susceptible to cardiovascular disease.

A study looked at the interaction of MASLD, AF, and HF. A Japanese nationwide epidemiological study including 3,279,918 adults aged above 20 years (median age 45 years, 57.6% men) that had the baseline metabolic function assessed in 2005–2021 were re-assessed after 1,160 ± 905 days. Diagnosis of fatty liver was based on the fatty liver index formulated by the Japan research group. 62,756 incident HF events and 15,408 incident AF events were recorded. Cox regression analysis was performed to study the association between MASLD, HF and AF. It was found that HRs for HF and AF, respectively, were 1.73 (95% CI: 1.69–1.76) and 1.51 (95% CI: 1.46–1.57) for MAFLD after adjusting for the cardiometabolic confounders (113), suggesting MASLD is positively associated with AF and HF. After a careful review of existing evidence on MASLD, AF and HF, we have found that MASLD, AF and HF are associated. However, further molecular studies and clinical studies are needed to examine the underlying causal relationships. Nevertheless, as MASLD is a relatively benign condition, we believe that initiating pharmacotherapies to treat MASLD early may prevent and treat AF and HF more effectively.

## Early treatment of MASLD to cardiac complications—AF & HF

AF and HF are public health concerns because treatment costs to manage AF and HF are high. Clinician-scientists have been studying the mechanisms of action of existing drugs because they hope to repurpose them to treat new conditions. As MASLD, AF and HF are closely related, the drugs that target the insulin-resistance pathway to treat MASLD may be able to treat AF and HF. In this section, we will focus on existing drugs that are used to treat MASLD (Table 3), and will discuss the practicability of using these drugs to treat AF and HF.

### Resmetirom

A new drug, resmetirom, was approved in March 2024 by the U.S. Food and Drug Administration (FDA) to treat non-cirrhotic MASH based on the study results of a phase 3 RCT in patients with MASH with liver fibrosis. Resmetirom is an oral, liver-directed, thyroid hormone receptor (THR) β-selective agonist in development. It mediates its action on nuclear thyroid hormone receptors (TRs) encoded by THR-α on chromosome 17 and THR-β on chromosome 3 genes. TR-1β is the dominant subtype in the liver, skeletal muscle and kidney (114). In the RCT that included 966 study subjects with biopsy-proven MASH, patients were randomized into a 1:1:1 ratio to either receive 80 mg or 100 mg once-daily resmetirom or placebo. Liver biopsy assessments were performed at the beginning and the end of the 52-week treatment period. Two primary endpoints were assessed: (1) MASH resolution, activity score reduced by ≥2 points with no worsening of fibrosis, and an improvement (reduction) in fibrosis by at least 1 stage with no further deterioration of MASLD activity score. It was found that 25.9% of those patients in the 80 mg and 29.9% of those in the 100 mg resmetirom groups, as compared with 9.7% of those in the placebo group (*p* < 0.001 for both comparisons with placebo) showed MASH resolution with no worsening of fibrosis. In addition, 4.2% of the patients in the 80 mg resmetirom group and 25.9% of those in the 100 mg resmethrin group, as compared with 14.2% of those in the placebo group (*p* < 0.001 for both comparisons with placebo) showed improvement by at least one stage with no worsening of the MASLD activity score.

### Pioglitazone

In the last decade, the drug class thiazolidinedione, which includes pioglitazone, is of popular use due to its relatively economical price and easy oral administration. It is indicated for treating T2D with or without comorbid MASLD. It acts as an insulin sensitizer to overcome insulin resistance. However, it has undesired side effects, including hypoglycemia, weight gain, fluid retention, skeletal fractures, hepatotoxicity, bladder cancer, atherosclerotic cardiovascular events and HF. Therefore, a declining prescribing pattern for pioglitazone has been observed after the newer anti-hyperglycemic drugs were introduced to the market (115, 116).

In a randomized, double-blinded, placebo-controlled trial of pioglitazone, patients were randomized to receive either pioglitazone or placebo for 6 months (117). A liver biopsy was performed before and after the treatment period. 26 patients receiving pioglitazone (mean age 51 ± 7 years, 53.8% men) and 21 patients receiving placebo (mean age 51 ± 10 years, 33% men) were examined. It was found that the pioglitazone group was associated with improvement in histologic findings about steatosis (*p* = 0.003), ballooning necrosis (*p* = 0.02), and inflammation (*p* = 0.008), when compared to the placebo group.

### Statin

Statin belongs to the drug class hydroxmethyglutaryl-CoA (HMG-CoA) reductase inhibitor (118). It is currently approved for hyperlipidemia and mixed dyslipidemia, primary dysbetalipoproteinemia, hypertriglyceridemia, atherosclerosis, primary prevention of atherosclerotic cardiovascular disease (ASCVD), secondary prevention in patients with clinical ASCVD, pediatric patients with familial hypercholesterolemia and adult patients with homozygous family hypercholesterolemia. Off-label uses include MASLD, cardiac allograft vasculopathy progression and prevention of contrast-induced acute kidney injury. Statin works by inhibiting the action of HKG-CoA reductase, an enzyme that converts HMG-CoA to mevalonate during cholesterol synthesis. In addition, statin also upregulates low-density lipoprotein (LDL) receptors and increases the LDL-cholesterol. Moreover, statin decreases the hepatic production of apo B-100 containing lipoproteins to lower cholesterol and triglyceride concentrations. MASLD is the excess accumulation of triglycerides in the liver; it is believed that the effect of statin on lowering triglycerides can treat patients with MASLD.

In a retrospective cohort study (119) including 1,238 patients (mean age 53.7 ± 14.2 years, 63.8% men) with MASLD, patients were assessed for their MASLD severity using the FIB-4 score. These patients were followed up for a mean of 3.3 years. It was found that 47% of patients were initiated on statins, and 18% of patients progressed to have high fibrosis risk. In the Cox regression analysis, statin intensity is associated with a lower risk to high-risk FIB-4 category (moderate statin intensity HR: 0.60, 95% CI: 0.42–0.84, and high statin intensity HR: 0.61, 95% CI: 0.42–0.88), after adjustment for sex, race, marital status, smoking status, body mass index, hypertension, diabetes, cardiovascular disease, hypothyroidism and chronic kidney disease. In another cohort study (*n* = 7,988, mean age = 53.0 ± 13.7 years, 41.8% men) involving 16 tertiary referral centres, statin usage is associated with lower long-term risk of all-cause mortality (adjusted HR: 0.233, 95% CI 0.127–0.426) and lower liver stiffness progression (HR: 0.542, 95% CI: 0.389–0.755).

### Metformin

Metformin has been the well-established first-line therapy for treating T2D for many years. It reduces liver gluconeogenesis, lowers intestinal absorption, and triggers insulin sensitivity to reduce blood glucose levels. This drug has a relatively safe medication profile and cardioprotective effects (120). In addition, the American Diabetes Association has now approved it for treating pre-diabetes. Apart from the FDA-approved indications, metformin is used to manage gestational diabetes, obesity/overweight, and polycystic ovarian syndrome.

In a 6-month metformin, double-blinded randomized placebo-controlled trial involving 24 study subjects (control group mean age: 44.3 ± 9.0 years, 80% men, treatment group mean age: 49.9 ± 12.8 years, 67% men) with biopsy-proven steatohepatitis in each arm, no significant differences between groups were observed for change in steatosis assessed histologically (121). However, the insignificant effect may be due to the small sample size.

### GLP-1 agonists

Glucagon-like peptide-1 (GLP-1) agonist is a relatively newer glucose-lower drug to treat T2D. GLP-1 and glucose-dependent insulinotropic polypeptide (GIP) are incretin hormones that trigger insulin secretion after ingesting dietary sugar. GLP-1 helps to delay gastric emptying and glucagon production from pancreatic *α*-cells. As well, GLP-1 decreases pancreatic β-cell apoptosis while increasing β-cell proliferation. It is currently indicated to treat obesity and T2D. Additional cardio-protective effects of GLP-1 agonists have been seen. In a randomized placebo-controlled trial named LEADER (Liraglutide and Cardiovascular Outcomes in Type 2 Diabetes) (122) that looked at the data of 9,340 patients with T2D who were randomized to receive either liraglutide 1.8 mg/d (or the maximum tolerated dose) or placebo, 3,692 (39.5%) history of MI/stroke and 3,083 (33.0%) atherosclerotic cardiovascular disease without MI/stroke events were observed. Comparing those with placebo using the Cox proportional hazard model, time to the first of the individual components of the 3-point MACE was consistently numerically reduced with liraglutide, including cardiovascular death (HR: 0.80, 95% CI: 0.63–1.02), nonfatal MI (HR: 0.83, 95% CI: 0.67–1.03), and nonfatal stroke (HR: 0.95, 95% CI: 0.71–1.27). Currently, off-label uses of GLP-1 agonists include metabolic-dysfunction-associated steatohepatitis (MASH) for those who do not achieve weight loss with lifestyle modification. However, whether GLP-1 agonist is beneficial against patients with MASLD without T2D has not been established.

In the multicentre, double-blinded, randomized and placebo-controlled 48-week liraglutide trial conducted in the UK from 2010–2013 (123), 52 patients with MASH were randomly assigned to either receive liraglutide (*n* = 26, mean age: 50 ± 11 years, 69% men in the treatment group or placebo, *n* = 26, mean age: 52 ± 12 years, 50% men). The primary outcome was defined as histological improvement in terms of resolution of steatohepatitis without worsening of fibrosis. Using the intention-to-treat analysis, patients with diabetes have better improvement (RR: 4.3, 95% CI: 1.0–17.7, *p* = 0.019). However, the use of GLP-1 agonists to treat patients without diabetes has not been investigated yet.

### SGLT-2 inhibitors

Sodium-glucose cotransporter-2 (SGLT-2) inhibitor is a newer anti-hyperglycemic drug class with cardio- and renal-protective effects. It was first approved by the FDA in 2013. SGLT-2 inhibitor works by suppressing the coupled glucose and sodium cotransporter reabsorption in the renal proximal tubule and elevating the urinary glucose and sodium excretion. Currently, SGLT-2 inhibitors are approved for multiple indications, including (1) glycemic control in T2D, (2) reduction of major adverse cardiovascular events in patients with T2D and established cardiovascular disease, (3) decrease the risk of cardiovascular hospitalization and death for HF in patients with HF with reduced ejection fraction, (4) reduction of the risk of estimated glomerular filtration rate decline and hospitalization in patients with chronic kidney disease at risk of progression, and (5) improvement of cardiovascular outcomes in patients with HF with preserved ejection fraction. Off-label use of SGLT-2 inhibitors includes (1) management of obesity in adjunct to GLP-1 agonists and (2) management of MASLD in patients comorbid with T2D. It is important to note that SGLT-2 inhibitors have low hypoglycemic risks due to their mechanism of action. It does not lower glucose levels without excess glucose in the circulation.

Given the mechanism of action of SGLT-2 inhibitors to reduce glucose reabsorption and insulin resistance, SGLT-2 inhibitors may play a significant role in reversing MASLD in patients. In an investigator-initiated, double-blind, randomized, placebo-controlled trial (RCT) conducted by the liver health census study group in 2021–2022, 98 patients (median age: 55.7 years, IQR: 49.5–63.4, 55.1% men) with MASLD without diabetes were randomized to receive either empagliflozin or placebo for 52 weeks, the Empagliflozin group had a more significant reduction in median MRI-PDFF compared to the placebo group (−2.49% vs. −1.43%, *p* = 0.025), with a nonsignificant trend of resolution of hepatic steatosis (44.9% vs. 28.6%, *p* = 0.094) (124).

## Emerging research and knowledge gaps

Patients with advanced liver fibrosis are at risk of developing AF and HF (113). Although we have mentioned that cardiokines, hepatokines, and adipokines work in autocrine, paracrine and endocrine manners to act as molecular signals to communicate between the heart and the liver, we still do not know the exact underlying pathophysiological molecular mechanisms linking MASLD, AF, and HF. Despite scientists have been employing the genomic approach to identify common genes involved in the disease progression of both MASLD and CVD, biomedical researchers have not yet been able to translate the research findings from GWAS into the wet lab. Therefore, scientists should continue to look for methods to track the molecular pathways linking the heart and the liver to discover molecular transmitters that cause diseases. Other than GWAS, proteomic analysis is another approach to discover and to investigate the underlying molecular mechanisms behind the association between MASLD and CVD. Recently, an advanced technology, named OLink Technology, has been made to identify the proteomic signatures in the given sample. It enables the high-throughput and multiplex of immunoassays using tiny volume of sample (125). Proteomic analysis is more accurate than genomic analysis because not all genes are transcribed into proteins due to epigenetic factors, such as environment, diet and xenobiotic use etc. Recessive genes have no phenotypic impact in daily life. Proteomic transmitters may serve as potential drug targets because if eliminated or reduced their expressions, the incidence of MASLD and CVD comorbidities and complications may be decreased.

In a randomized clinical trial (124) that targeted patients with MASLD without diabetes, the SGLT-2 inhibitor did not achieve the goal of resolving hepatic steatosis because the investigators did not specifically select to include patients with more advanced stages of MASLD. 86.7% of the study subjects only had mild steatosis without liver fibrosis; the median baseline liver stiffness measurement was 5.3 kPa. These patients might not have severe insulin resistance that would benefit from SGLT-2 inhibitors, as the proposed mechanism of action for SGLT-2 inhibitors to treat MASLD is through reducing insulin resistance. Therefore, whether SGLT-2 inhibitors may help treat patients with more advanced stages of MASLD is not addressed in this RCT and remains uncertain. Given the mechanism of action of SGLT-2 inhibitors to reduce insulin resistance through decreasing glucose and sodium reabsorption, SGLT-2 inhibitors may play a significant role in reversing advanced liver fibrosis. There is currently no existing trial looking at the effectiveness of SGLT-2 inhibitors to reduce insulin resistance to treat patients with more advanced stages of liver fibrosis without T2D. Apart from SGLT-2 inhibitors and other existing drugs, some therapeutics, such as Efruxifermin (126), are under investigation. Efruxifermin is a long-acting Fc-FGF21 fusion protein for the treatment of NASH. A phase 2 RCT was conducted in patients with NASH cirrhosis (127); 80 patients were randomly assigned to receive either placebo, 28 mg, 50 mg or 70 mg efruxifermin subcutaneous injection weekly for 16 weeks. In the complete analysis set, efruxifermin significantly reduced liver fat in patients with MASH and F1-F3 fibrosis. The effects were sustainable after 16 weeks of treatment and might modulate MASH progression.

## Conclusion

Signalling transmitters mediate the heart-liver cross-talk, inducing inflammation and oxidative stress and modulating the immune system. This subsequently causes MASLD, AF, and HF. As these diseases influence each other, existing drugs that target the cardiometabolic pathways to reduce insulin resistance, fat uptake, and inflammation to treat MASLD are potential agents to treat and prevent cardiac complications, such as AF and HF.
